# Croup Cured by Cauterizing the Larynx with Nit. Arg.

**Published:** 1847-07

**Authors:** Wm. N. Blakeman


*Two Cases of Croup cured by Cauterizing the Larynx with a Solution of Nitrate of Silver.*—By Wm. N. Blakeman, M. D. On the 10th Nov. 1847, I was called to see a child of Mr. A. about two years old, very fat, large of his age, and of leucophlegmatic temperament. I saw him at 10 o'clock in the evening, five hours after the commencement of the disease, with a hot, dry skin, quick pulse, great restlesness, laborious breathing, and the hoarse barking or crowing sound peculiar to croup. The family had, previous to my arrival, given freely of Coxe's hive syrup.

I gave tinct. sang., comp. syrup scillæ, with pulv. ipecac., which caused vomiting, but no relief to the patient. At 3 o'clock on the morning of the 11th, I gave six grain prot. chlor. hyd., and after waiting two hours, began with above mixture, to which I added five grains of tart. antim.; more free vomiting was produced, and a copious discharge from the bowels, at 3 o'clock, but without any mitigation of a single symptom. I then stopped using the above mixture, and gave per-sulph. of mer., in doses of qu. grain, the second dose to be given in half an hour after the first, and then at intervals of an hour. The child drank freely of warm water, and vomited some after each repetition of the medicine, but none of that peculiar, heavy, glairy substance, which is the secretion of this specific inflammation. At 5 o'clock, P. M., the remedies having done no good, and with the symptoms of suffocation becoming alarming, I resolved to try the effect of cauterizing the larynx with a solution of nitrate of silver, a drachm to an ounce of water.

The application was somewhat difficult, and the dyspnœa very great. A quantity of the thick tenacious substance was brought away by the sponge, &c., a large quantity by vomiting, which followed.

After waiting ten minutes, I made a second application, bringing away a larger quantity of membranous matter on the sponge than before, and a much more copious discharge accompanied the vomiting, caused by the application.

The disease now seemed to be arrested, as very great relief was apparent to all the family. The breathing was less laborious, the crowing sound less sharp, and the child more quiet.

I saw the boy at half-past 10 o'clock, same evening, five hours and a half after the first application; he had improved in all the symptoms, breathing decidedly better, the barking sound heard only at intervals, and he had asked for drink.

I now made a third application of the same solution, which brought as before, on the sponge, some thick tenacious matter, differing from the first in being of a yellow color. The boy vomited *several* times after *this* application, each time throwing off a large quantity of the same yellow-colored, thick substance, so tough that it could be raised from the bowl by the fingers. Soon after the vomiting ceased the child was so much better he fell asleep, in which situation I left him, with directions to be called if required before morning.

12th, 7 o'clock, A. M., I found him sitting in the bed calling for food; he had slept pretty well, asking for drink occasionally, a slight hoarseness left, for which he required no further treatment.

Case II.—I was called on the 20th of January, at 12 o'clock at night, to see a boy six years old, of sanguine temperament, and florid complexion, who was taken about two hours before with croup. The pulse quick, skin hot and dry, the breathing hurried and difficult, the crowing noise loud, and the child very restless. I determined that the remedy used last in the former case should be first in this. I made two applications of the same solution used in the former case. Some tough phlegm came away on the sponge, and free vomiting followed, which relieved the patient so that he fell asleep.

21st, 7 o'clock, A. M. The boy has slept well all night, and says he is quite well, only a little hoarse.—*N. Y. Medical and Surgical Reporter.*

